# Can video head impulse testing aid differential assessment of suspected vestibular migraine attacks?

**DOI:** 10.3389/fneur.2026.1926662

**Published:** 2026-08-17

**Authors:** Yufei Wang, Yonghui Zhang, Daopei Zhang

**Affiliations:** 1 Department of Encephalopathy, The First Affiliated Hospital of Henan University of Chinese Medicine, Zhengzhou, China; 2 Henan Institute of Vertigo Research, Henan University of Chinese Medicine, Zhengzhou, China

**Keywords:** differential diagnosis, ictal phase, refixation saccades, subjective visual vertical, vestibular migraine, video head impulse test

## Abstract

Vestibular migraine is a clinically defined disorder characterized by recurrent vestibular symptoms associated with migraine features, and its acute attacks may be difficult to distinguish from peripheral vestibular disorders such as acute unilateral vestibulopathy/vestibular neuritis, Ménière’s disease, and vestibular schwannoma. This review synthesizes current evidence on the clinical interpretation and proposed pathophysiological context of video head impulse test findings during acute vestibular migraine episodes, with particular attention to vestibulo-ocular reflex gain, refixation saccades, and subjective visual vertical abnormalities. Available evidence suggests that vestibulo-ocular reflex gain is often preserved during acute vestibular migraine, although variable or transient reductions have also been reported, limiting its diagnostic specificity when used alone. Refixation saccades, including covert, bilateral small-amplitude, or occasionally unilateral high-amplitude patterns, have also been reported during acute vestibular migraine. However, these findings are non-specific and may also be associated with peripheral vestibular dysfunction, age-related variability, compensatory responses, gaze-fixation instability, or technical artifacts. When recording quality is adequate, their distribution and temporal evolution may provide adjunctive information, but they should not be used independently to infer a specific central mechanism or establish the diagnosis of vestibular migraine. Subjective visual vertical testing may provide complementary information on graviceptive perceptual imbalance that is not fully captured by vestibulo-ocular reflex gain. Because VM is diagnosed on clinical grounds, vHIT and SVV findings should be interpreted as non-specific adjunctive data within the Bárány Society and International Headache Society diagnostic framework and in conjunction with the clinical history and complementary vestibular and audiological assessments. Accordingly, vHIT and SVV may contribute to a multimodal differential assessment during VM attacks, but they do not define a specific pathophysiological subtype or replace the clinical diagnosis of VM.

## 1 Introduction

Vestibular migraine (VM) is a clinically defined disorder diagnosed according to criteria jointly developed by the Bárány Society and the International Headache Society (1–3). Definite VM requires at least five episodes of moderate or severe vestibular symptoms lasting 5 min to 72 h, a current or previous history of migraine with or without aura, one or more migraine features during at least 50% of vestibular episodes, and symptoms not better accounted for by another vestibular or headache disorder. This condition not only severely impairs patients’ daily activities and quality of life but is also frequently misdiagnosed as other peripheral vestibular disorders, such as acute unilateral vestibulopathy/vestibular neuritis (AUVP/VN) and Ménière’s disease. The video head impulse test (vHIT), a core technology for objectively and quantitatively assessing vestibulo-ocular reflex (VOR) function, has become an important tool in vestibular examination owing to its portability and accuracy. It provides quantitative information on high-frequency VOR function that may contribute to the clinical assessment and differential diagnosis of vestibular disorders. Nevertheless, existing studies have largely focused on vHIT application during the interictal phase of VM, leaving its utility during acute attacks insufficiently studied. Accordingly, this review examines vHIT findings during acute VM attacks, with emphasis on VOR gain, refixation saccades, complementary vestibular assessments, and differential diagnosis. Its aim is to clarify the adjunctive role and limitations of vHIT in the clinical assessment of suspected acute VM rather than to propose vHIT as an independent diagnostic test.

### 1.1 Literature search and selection

This article was designed as a narrative review supported by a structured literature search. The methodological organization and reporting of this review were guided by the six quality domains of the Scale for the Assessment of Narrative Review Articles (SANRA), including justification of the review’s importance, a clear statement of its aims, description of the literature search, appropriate referencing of key statements, balanced scientific reasoning, and appropriate presentation of relevant evidence. Relevant publications were identified through searches of PubMed/MEDLINE, Web of Science Core Collection, and Scopus from database inception to June 1, 2026 (4). Search concepts included “vestibular migraine,” “migrainous vertigo,” “acute attack,” “ictal phase,” “video head impulse test,” “vHIT,” “vestibulo-ocular reflex gain,” “refixation saccades,” “corrective saccades,” “catch-up saccades,” “covert saccades,” “overt saccades,” “subjective visual vertical,” “vestibular-evoked myogenic potentials,” “caloric testing,” “suppression head impulse paradigm,” and “pupillary hippus.” Reference lists of relevant articles were also screened to identify additional publications.’

Peer-reviewed original clinical studies, consensus documents, systematic reviews, and clinically relevant narrative reviews were considered. Methodological studies were included when they addressed vHIT acquisition, gain calculation, saccade interpretation, biological variability, or technical artifacts. Preclinical or mechanistic studies were considered only when they provided relevant neuroanatomical or pathophysiological context and were not used to establish the diagnostic accuracy or clinical specificity of vHIT findings. Publications were prioritized according to their direct relevance to acute or ictal vestibular migraine, complementary vestibular assessment, and differential diagnosis from acute unilateral vestibulopathy/vestibular neuritis, Ménière’s disease, and vestibular schwannoma. Conference abstracts without a full peer-reviewed report, non-peer-reviewed sources, and publications without direct relevance to the review questions were excluded.

Because the objective was to provide an interpretive and clinically focused synthesis rather than to estimate a pooled diagnostic effect, the evidence was synthesized narratively, and no meta-analysis or formal risk-of-bias assessment was performed. The anonymized illustrative clinical cases were not included in the literature search, study selection, or evidence synthesis.

## 2 Pathophysiological context for video head impulse testing during vestibular migraine attacks

**Figure 1:**
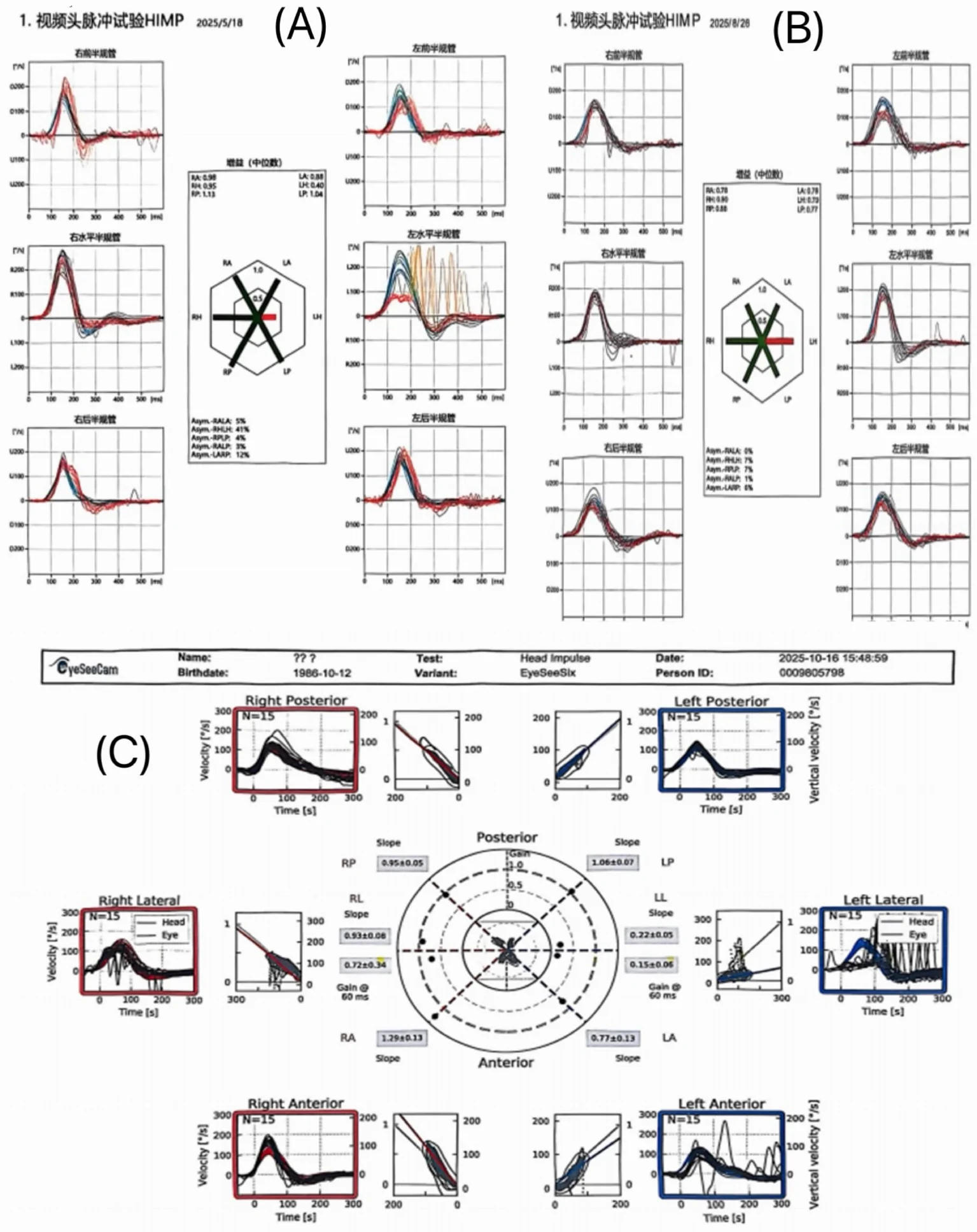
Illustrative clinical case: Transiently reduced horizontal-canal VOR gain with overt refixation saccades **(A)** At the initial visit on May 18, 2025, the video head impulse test (vHIT) showed a reduced vestibulo-ocular reflex (VOR) gain of 0.40 in the left horizontal semicircular canal. After the head impulse, prominent, high-velocity overt refixation saccades appeared in the direction opposite to the head movement **(B)** At the follow-up visit on August 28, 2025, the VOR gain in the left horizontal semicircular canal had improved to 0.79, and the amplitude of the overt refixation saccades was reduced **(C)** At the subsequent visit on October 17, 2025, VOR gains and eye-movement traces across all bilateral semicircular canals were stable, and the previously observed overt refixation saccades had largely resolved. Panels A and B were acquired using the VertiGoggles ZT-VNG-II system, whereas panel C was acquired using the EyeSeeCam system.

**Figure 2:**
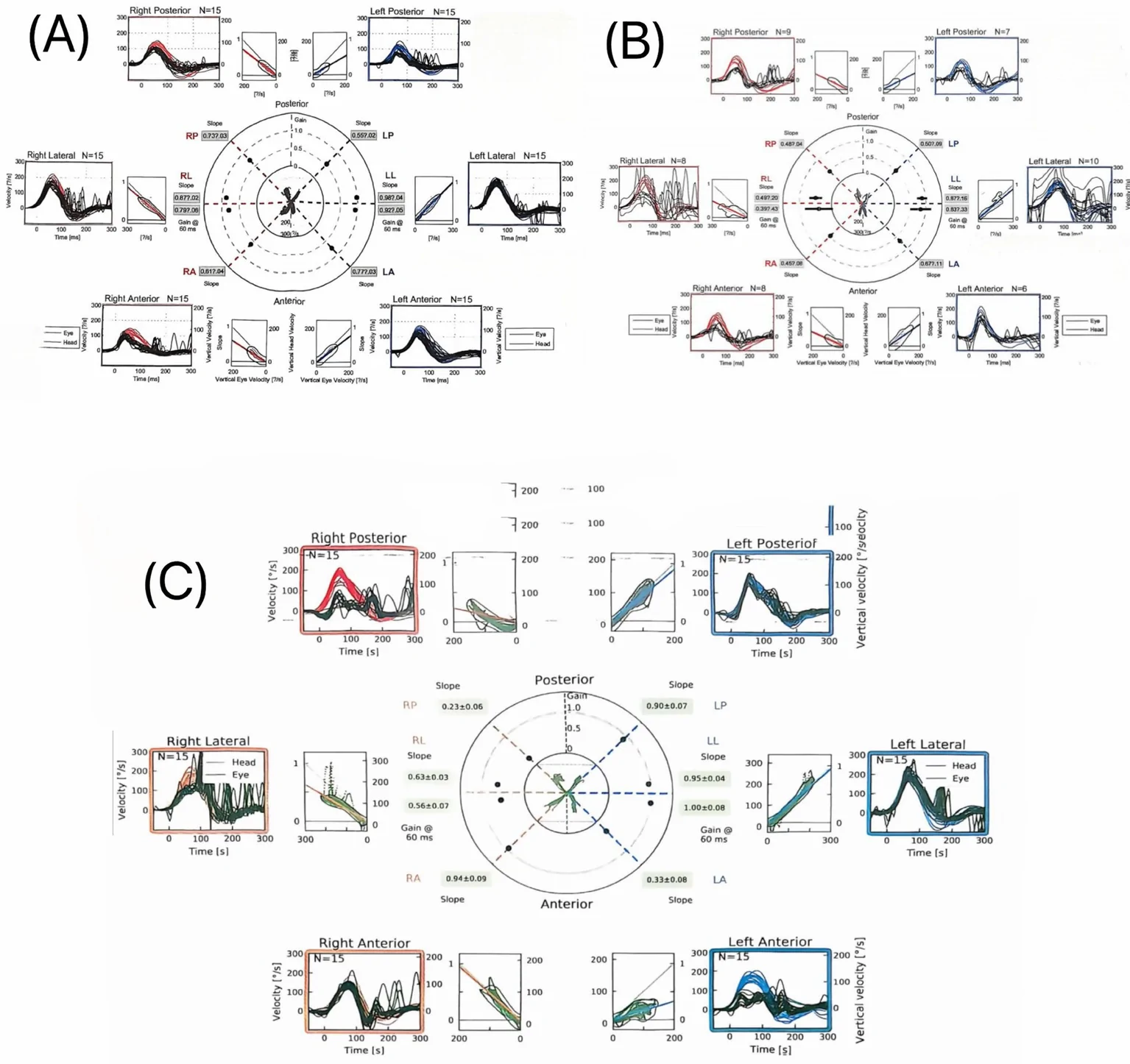
Illustrative clinical case: Bilateral small-amplitude covert refixation saccades with preserved VOR gain. **(A)** On July 5, 2022, vestibulo-ocular reflex (VOR) gains for all semicircular canals bilaterally remained within the device-specific reference ranges. Small-amplitude, scattered covert refixation saccades were present in the eye-velocity traces of the bilateral horizontal semicircular canals. **(B)** On April 2, 2025, VOR gains remained preserved across all semicircular canals. The amplitude of the bilateral covert refixation saccades had decreased, and their distribution had become more regular. **(C)** By the follow-up on May 8, 2025, VOR gains remained preserved, and the previously observed bilateral covert refixation saccades had largely resolved. All video head impulse test recordings in this figure were acquired using the EyeSeeCam system.

**Figure 3:**
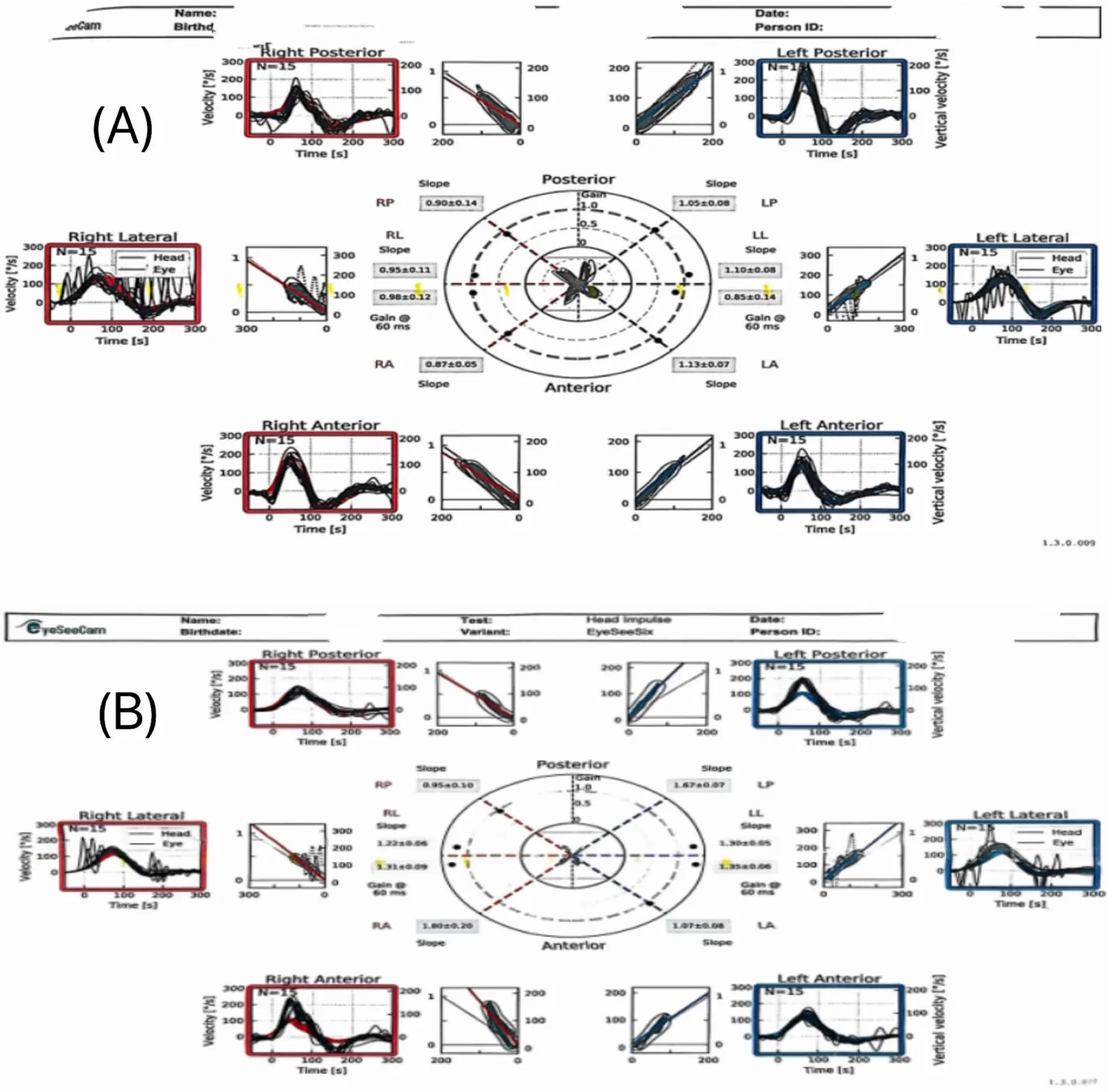
Illustrative clinical case: Unilateral high-amplitude overt refixation saccades with preserved VOR gain **(A)** On October 23, 2025, vestibulo-ocular reflex (VOR) gains across the bilateral semicircular canals remained within the device-specific reference ranges. Prominent, relatively regular, high-amplitude overt refixation saccades were observed in the eye-velocity trace of the left horizontal semicircular canal **(B)** At follow-up on October 28, 2025, VOR gains remained preserved, whereas the previously observed unilateral high-amplitude overt refixation saccades were markedly attenuated and had largely resolved. All video head impulse test recordings in this figure were acquired using the EyeSeeCam system.

**Figure 4:**
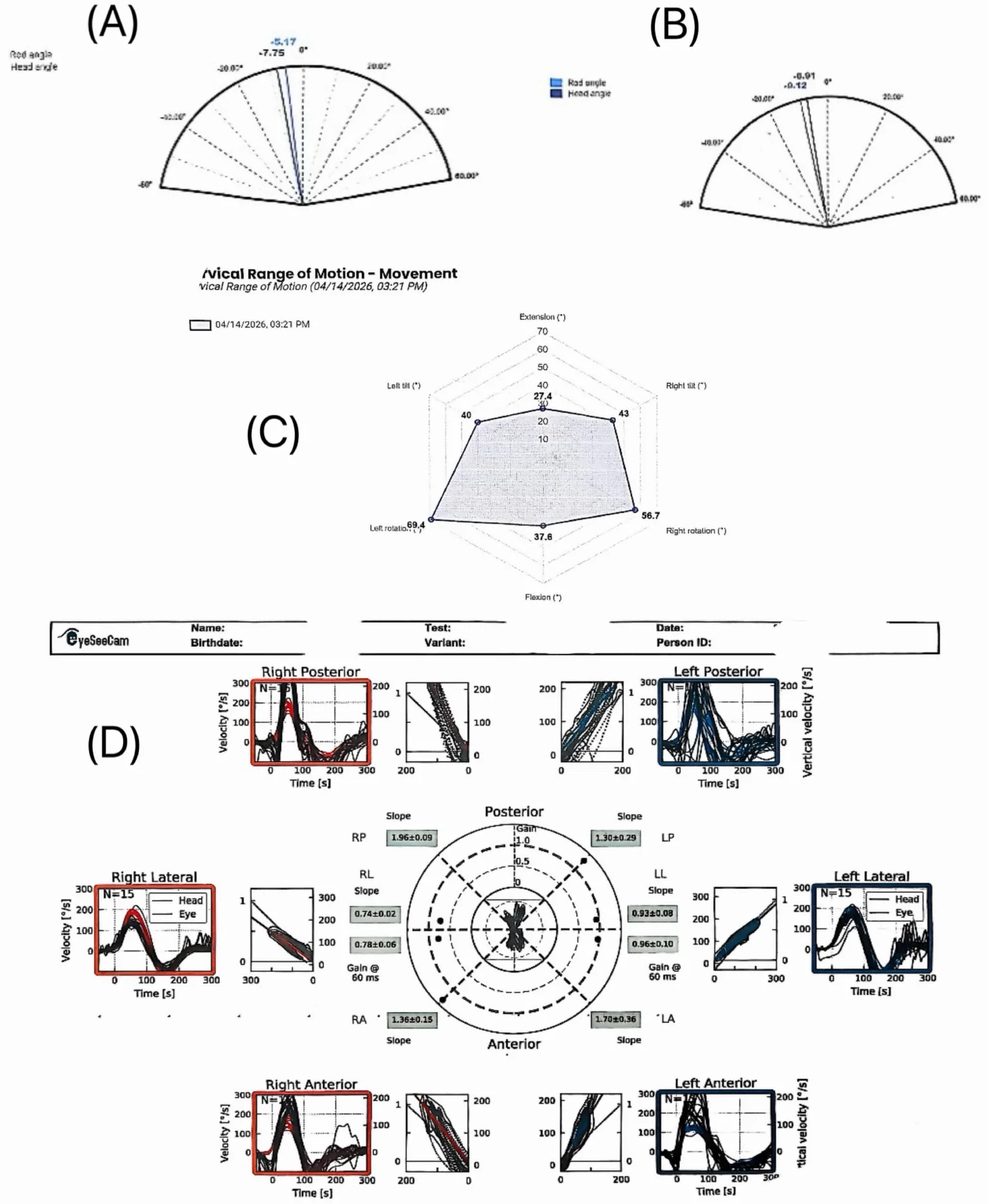
Illustrative clinical case: Abnormal Subjective Visual Vertical with preserved vHIT findings. **(A)** At the clinical visit on April 14, 2026, Subjective Visual Vertical (SVV) testing showed a deviation of −7.75°. **(B)** Head-posture assessment demonstrated abnormal angular alignment. **(C)** Cervical range-of-motion assessment recorded active neck flexion, extension, left and right lateral flexion, and left and right rotation, with restriction predominantly affecting extension and rotation. **(D)** On April 15, 2026, video head impulse testing (vHIT) showed preserved vestibulo-ocular reflex (VOR) gains and stable eye-velocity traces in the bilateral semicircular canals, without evident refixation saccades. All video head impulse test recordings in this figure were acquired using the EyeSeeCam system.

The video head impulse test (vHIT) is widely used in clinical practice. Commercial video head impulse test (vHIT) systems may differ in their goggle configuration, eye-tracking hardware, head-motion sensors, and gain-calculation methods (5, 6). The illustrative recordings shown in Figure 1 were obtained using two different systems. Panels A and B were acquired using a VertiGoggles ZT-VNG-II system (Shanghai ZEHNIT Medical Technology Co., Ltd., Shanghai, China), which uses binocular high-speed infrared video recording and integrated head-motion tracking. Panel C and the recordings shown in Figures 2–4 were acquired using an EyeSeeCam system (Interacoustics A/S, Middelfart, Denmark). The EyeSeeCam system uses a lightweight goggle with an interchangeable monocular USB infrared camera to record eye movements and an integrated inertial measurement unit to record head movements, with a sampling rate of 220 Hz. Data were acquired and analyzed using the respective manufacturer-provided software. Because hardware configurations and gain-calculation methods may differ between systems, vestibulo-ocular reflex gain values were interpreted according to the device-specific reference ranges, and no direct quantitative comparison between the two platforms was intended (7–9). The test primarily characterizes the high-frequency function of the semicircular-canal vestibulo-ocular reflex during brief, passive, high-velocity and high-acceleration head impulses and should not be interpreted as a comprehensive measure of overall vestibular function. The rapid onset component of the head-impulse response is thought to depend predominantly on fast type I hair-cell–calyx units and irregular canal afferents in the central region of the crista. However, this physiological distinction is not absolute, because sustained and transient canal pathways contribute jointly to the compensatory eye movement; therefore, vHIT should not be regarded as a selective test of type I hair cells. During the brief, high-acceleration impulses used in vHIT, the early compensatory eye response is weighted toward the short-latency semicircular canal–vestibular nucleus–ocular motor pathway, whereas visual tracking contributes little because of the short duration of the stimulus (10, 11). The velocity-storage mechanism is an indirect central vestibular integrator that prolongs and spatially organizes vestibular and optokinetic responses. It should therefore not be described as being deactivated by vHIT, although its relative contribution to the brief high-frequency response is limited. Distinct from velocity storage, the ocular motor velocity-to-position neural integrator is a brainstem–cerebellar network that converts eye-velocity commands into tonic eye-position signals required for stable gaze (12, 13). Dysfunction of this gaze-holding network may produce centripetal eye drift, gaze-evoked nystagmus, or rebound nystagmus and may therefore affect ocular stability independently of peripheral semicircular-canal function (14–16). Central ocular motor abnormalities have been reported during both ictal and interictal phases of vestibular migraine, and gaze-evoked nystagmus has been observed in some patients. These observations make transient dysfunction of central gaze-holding networks a plausible contributor to selected ocular motor manifestations of vestibular migraine. However, current evidence does not establish that neural-integrator dysfunction determines the clinical type or severity of vestibular migraine attacks or directly generates a specific vHIT refixation-saccade pattern. Accordingly, gaze-holding abnormalities should be assessed directly by bedside ocular motor examination or video-oculography and should not be inferred from vHIT traces alone. Pathological changes at different levels of this pathway may alter VOR function and produce abnormal vHIT findings. However, vHIT findings do not independently localize the affected level or determine whether the underlying disorder is peripheral or central.

The pathophysiology of vestibular migraine (VM) remains incompletely understood and has been proposed to involve interactions between trigeminal nociceptive and vestibular networks. Neuroanatomical and neurophysiological observations suggest reciprocal connections between the spinal trigeminal nucleus caudalis and the vestibular nuclei, providing a possible substrate through which trigeminovascular activation may influence vestibular processing during migraine attacks (17). In parallel, calcitonin gene-related peptide (CGRP) and other migraine-related neurochemical changes may contribute to altered sensory processing, although their direct relationship to specific vHIT waveform morphologies has not been established (18). Clinical studies indicate that VOR gain is often preserved in VM, while dissociation between normal vHIT findings and abnormal caloric responses may occur in VM, Ménière’s disease, and other vestibular disorders; therefore, these findings are not disease-specific (19, 20). Accordingly, vHIT should be regarded as an assessment of high-frequency semicircular-canal VOR function that may contribute to differential evaluation when interpreted alongside the clinical history and complementary vestibular tests. It cannot independently confirm VM, establish a specific central mechanism, or exclude all peripheral vestibular disorders.

## 3 Video head impulse test (vHIT) and its Core indicators for evaluating vestibular migraine

### 3.1 Vestibulo-ocular reflex gain

Vestibulo-ocular reflex (VOR) gain quantifies the relationship between compensatory eye velocity and head velocity during a brief head impulse and is a principal quantitative outcome of the video head impulse test (vHIT) for characterizing high-frequency semicircular-canal VOR function (6). Because commercial vHIT systems may use different gain-calculation algorithms, including instantaneous, regression-based, or area-under-the-curve methods, absolute gain values are not necessarily interchangeable across devices (5, 9). Normative VOR gain values vary according to the vHIT device, gain-calculation algorithm, semicircular-canal plane, head-impulse characteristics, and laboratory reference population. Therefore, VOR gain should be interpreted using device- and laboratory-specific reference ranges rather than a single universal normal interval or pathological cutoff. Approximate lower limits of 0.8 for the horizontal canals and 0.7 for the vertical canals are commonly used in clinical practice and research, but these values should be regarded as practical reference points rather than universal diagnostic thresholds (21–23). For comparison, the Bárány Society diagnostic framework for bilateral vestibulopathy includes a bilateral horizontal angular VOR gain below 0.6 at head velocities of approximately 150–300°/s as one laboratory criterion (24). This disease-specific threshold should not be interpreted as a universal cutoff for unilateral vestibular hypofunction, vestibular migraine, or general vHIT abnormality (25–27).

Reduced VOR gain may indicate impaired high-frequency semicircular-canal function or dysfunction along the associated VOR pathway, but it does not independently identify the lesion site, establish the underlying disease, or diagnose vestibular migraine. Interpretation should also consider the reproducibility and quality of the head impulses, the presence and characteristics of refixation saccades, recording artifacts, and complementary vestibular findings (16, 28, 29).

### 3.2 Refixation saccades

Rapid eye movements that restore fixation on a visual target during or after a head impulse are commonly described as refixation, corrective, or catch-up saccades. For terminological consistency, this review uses “refixation saccades” as the umbrella term. Based on their timing relative to the head impulse, refixation saccades are classified as covert when they occur during head movement and overt when they occur after head movement (8, 30).

Refixation saccades are not specific to a particular lesion site or disease mechanism. They are commonly observed when vestibulo-ocular reflex-mediated gaze stabilization is insufficient or unstable, particularly in peripheral vestibular hypofunction, but they may also occur when VOR gain remains within the device-specific reference range. Their occurrence and measured characteristics may additionally be influenced by vestibular compensation, age-related variability, central oculomotor or gaze-stabilization abnormalities, and technical artifacts. Therefore, interpretation should integrate VOR gain, saccade reproducibility, timing, amplitude and direction, recording quality, and the overall clinical context (8, 31–34). Beyond the covert–overt distinction, refixation saccades may also be described according to their latency from head-impulse onset and their temporal dispersion across repeated impulses. “Early” and “late” saccades are relative latency categories, and the proposed cutoffs may vary according to the testing protocol, stimulus predictability, and analytical method. A gathered pattern refers to saccades recurring within a relatively narrow latency window across repeated impulses, whereas a scattered pattern reflects greater trial-to-trial temporal dispersion; this temporal organization may be quantified using measures such as the Perez–Rey score. Because saccade latency and clustering may be influenced by the stage of vestibular compensation as well as by head-impulse characteristics, visual conditions, recording quality, device-specific algorithms, and individual variability, these features should not be used alone to determine the age, severity, or cause of a vestibular lesion (35, 36).

#### 3.2.1 Overt saccades

Overt refixation saccades occur after the head impulse has ended and return the eyes toward the visual target. They may therefore be visible during the bedside head impulse test and appear on vHIT recordings as eye-velocity peaks after the completion of head movement. Overt refixation saccades commonly accompany insufficient VOR-mediated gaze stabilization, particularly in peripheral vestibular hypofunction, but their presence alone does not establish marked vestibular loss. They may also occur when VOR gain remains within the device-specific reference range, while their amplitude and detection may be influenced by age, recording variability, device-related differences, and technical artifacts (8, 15, 21, 30–32, 34, 37). Although overt refixation saccades may be prominent after acute unilateral vestibular loss, their latency and temporal dispersion can vary, they may coexist with covert saccades, and they may persist during follow-up even after VOR gain has partially or completely recovered. Accordingly, a late or scattered overt-saccade pattern should not be interpreted in isolation as a direct marker of lesion age or severity.

**Figure 5:**
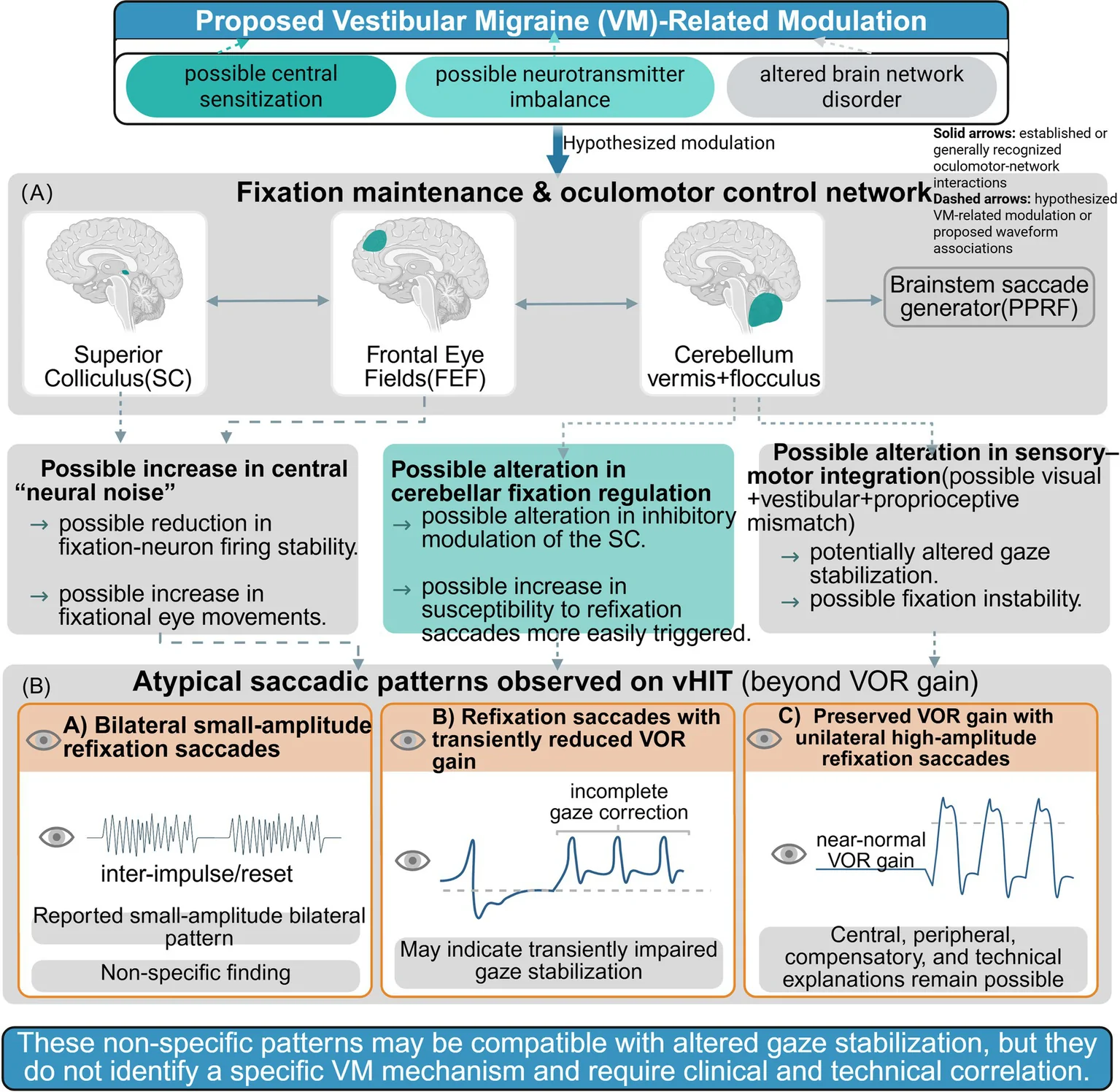
Conceptual model of possible vestibular migraine–related modulation of fixation and oculomotor control and non-specific saccadic patterns reported on Video Head Impulse Testing. **(A)** The Superior Colliculus, Frontal Eye Fields, Cerebellar Vermis/Flocculus, and Paramedian Pontine Reticular Formation are established or generally recognized components of fixation and saccadic control. Possible central sensitization, neurotransmitter dysregulation, and altered brain-network integration are shown as hypothesized modulators of these networks in vestibular migraine rather than as established causes of specific eye-movement abnormalities. **(B)** Schematic examples of non-specific vHIT patterns discussed in this review include bilateral small-amplitude refixation saccades, refixation saccades with transiently reduced vestibulo-ocular reflex gain, and unilateral high-amplitude refixation saccades with preserved vestibulo-ocular reflex gain. These patterns may be compatible with altered gaze stabilization, but they do not identify a specific pathophysiological mechanism of vestibular migraine. Peripheral vestibular dysfunction, vestibular compensation, age-related and interindividual variability, and technical artifacts such as goggle slippage, calibration error, inadequate pupil tracking, and suboptimal head-impulse quality should also be considered. Solid arrows indicate established or generally recognized interactions within the fixation and oculomotor control network, whereas dashed arrows indicate hypothesized VM-related modulation or proposed associations with vHIT patterns.

Overt refixation saccades have also been reported in patients with VM, but the available evidence does not establish them as a VM-specific finding or as a marker of diminished central inhibition. They should therefore be interpreted as non-specific findings together with VOR gain, recording quality, examination timing, complementary vestibular findings, and the overall clinical context (38, 39)(Figure 5).

#### 3.2.2 Covert saccades

Covert refixation saccades may occur as part of an adaptive response to insufficient VOR-mediated gaze stabilization and may be observed during both acute and compensated stages of peripheral vestibular loss. Earlier or shorter-latency covert saccades, particularly when they form a more gathered temporal pattern across repeated impulses, may be compatible with adaptive or predictive compensation. However, neither their latency nor their degree of temporal clustering independently dates the lesion, quantifies its severity, or identifies its underlying cause (8, 30, 33).

Covert refixation saccades have been reported in patients with vestibular migraine (VM) (38, 39). Although central sensitization is an important component of migraine pathophysiology (40, 41), available evidence does not demonstrate that trigeminal–vestibular hyperexcitability directly generates covert refixation saccades or that these saccades specifically reflect an abnormal velocity-storage time constant in VM. Available evidence likewise does not establish that the same proposed hyperexcitability directly causes an increase in vHIT-derived VOR gain. Refixation saccades and VOR gain should therefore be interpreted as related but distinct measures of gaze stabilization. Subclinical peripheral vestibular dysfunction, vestibular compensation, age-related variability, device-related differences, and technical artifacts remain alternative explanations. Covert refixation saccades should therefore be interpreted as non-specific adjunctive findings together with VOR gain, recording quality, complementary vestibular assessments, and the overall clinical context (8, 15, 30–32).

#### 3.2.3 Technical and biological factors affecting saccade interpretation

Before refixation saccades are considered clinically meaningful, the technical quality of the vHIT recording should be carefully reviewed. Goggle slippage, inadequate calibration, blinking, loss of pupil tracking, excessive facial movement, inappropriate head-impulse velocity or direction, and device-specific processing algorithms may alter VOR gain or generate spurious eye-velocity peaks. Device-related differences may also affect both measured gain and the detection and characterization of refixation saccades (15, 21, 32).

Biological and interindividual variability should also be considered. Aging may influence refixation-saccade amplitude, while saccade latency, amplitude, frequency, and clustering may vary among individuals and across repeated examinations. Therefore, only reproducible saccades observed across technically adequate impulses should be included in clinical interpretation. Even when reproducible, these findings remain non-specific and should be interpreted together with VOR gain and complementary vestibular assessments (21, 22, 31, 32).

### 3.3 Complementary role of HIMP and SHIMP

The conventional Head Impulse Paradigm (HIMP) requires fixation on an earth-fixed target. When high-frequency vestibulo-ocular reflex (VOR)-mediated gaze stabilization is insufficient, compensatory refixation saccades are generated to return gaze to the target. In the Suppression Head Impulse Paradigm (SHIMP), the visual target moves with the head. When vestibular function is preserved, the initial VOR drives the eyes away from the head-fixed target, followed by an anti-compensatory saccade in the direction of head rotation. Accordingly, compensatory saccades during HIMP generally indicate insufficient VOR-mediated gaze stabilization, whereas the presence and characteristics of anti-compensatory saccades during SHIMP provide complementary information on residual high-frequency vestibular function and vestibulo-saccadic interaction. HIMP and SHIMP may therefore be used together to characterize high-frequency VOR function more comprehensively (42, 43).

Although SHIMP engages visually guided VOR suppression, its gain and saccadic responses should not be interpreted as isolated measures of central suppression. They may also be influenced by residual peripheral VOR function, saccade generation, stimulus predictability, visual conditions, vestibular compensation, recording quality, and device-specific factors. Accordingly, disagreement between HIMP and SHIMP saccadic patterns may prompt further assessment of central ocular-motor and visual–vestibular integration, but it does not by itself establish a central lesion or distinguish vestibular migraine from peripheral vestibular disease. Available studies in vestibular migraine have reported variable findings: one cohort demonstrated predominantly preserved HIMP and SHIMP gains, whereas a smaller case–control study reported absent, scattered, or asymmetric anti-compensatory saccades. These observations have not established an acute-attack-specific or vestibular-migraine-specific SHIMP phenotype. SHIMP has also been shown to be feasible and potentially informative in pediatric vestibular assessment, including in children with vestibular migraine; however, the available pediatric evidence is heterogeneous and does not validate SHIMP as a mandatory or vestibular-migraine-specific diagnostic test. SHIMP should therefore be considered a complementary examination when available, while the diagnosis of vestibular migraine remains clinical and requires integration with the detailed history and other vestibular, ocular-motor, auditory, and neurological findings (44–46).

## 4 Characteristics of video head impulse test during vestibular migraine attacks

Previous studies have largely focused on applying the video head impulse test (vHIT) to diagnose vestibular migraine (VM) during the interictal period, yielding several findings. These include predominantly normal gain in most patients, along with saccades that are bilateral, small in amplitude, and scattered in distribution (39). As research has progressed, notable differences in vHIT results have emerged between the ictal and interictal phases of VM, and the variation in core parameters across these periods has challenged conventional understanding.

### 4.1 Predominantly preserved or variable Vestibulo-ocular reflex gain

Vestibulo-ocular reflex (VOR) gain is a principal quantitative parameter of the video head impulse test (vHIT), but available studies do not demonstrate a consistent attack-related reduction in patients with vestibular migraine (VM). Koç et al. (47) reported lower mean VOR gains across all semicircular canals in patients examined during VM attacks than in healthy controls, although the differences did not reach statistical significance. Patel et al. (48) found that horizontal-canal VOR gain remained within the reference range during acute vertigo attacks and did not differ significantly from that of controls. The findings reviewed by Chen et al. (49) and the repeated-measures observations of Batinović et al. (50) were broadly consistent with largely preserved VOR gain and no uniform phase-dependent change. Taken together, preserved or mildly variable VOR gain appears common in VM, but it is neither universal nor disease-specific. One interictal case–control study reported higher vertical-canal VOR gains in patients with VM than in healthy controls, whereas horizontal-canal gains were similar between groups (20). However, this isolated observation has not been consistently reproduced in ictal or repeated-measures studies and has not been established as a characteristic feature of VM attacks (47–50). Although altered central modulation or adaptation of the VOR could theoretically influence gain, measured values may also be affected by calibration, goggle stability, head-impulse characteristics, and device-specific gain-calculation algorithms (15, 21). At present, no direct evidence demonstrates that trigeminal–vestibular hyperexcitability causes increased VOR gain in VM (51). Accordingly, an isolated high VOR gain should not be interpreted as a marker of central sensitization or used independently to support the diagnosis of VM.

**Figure 6:**
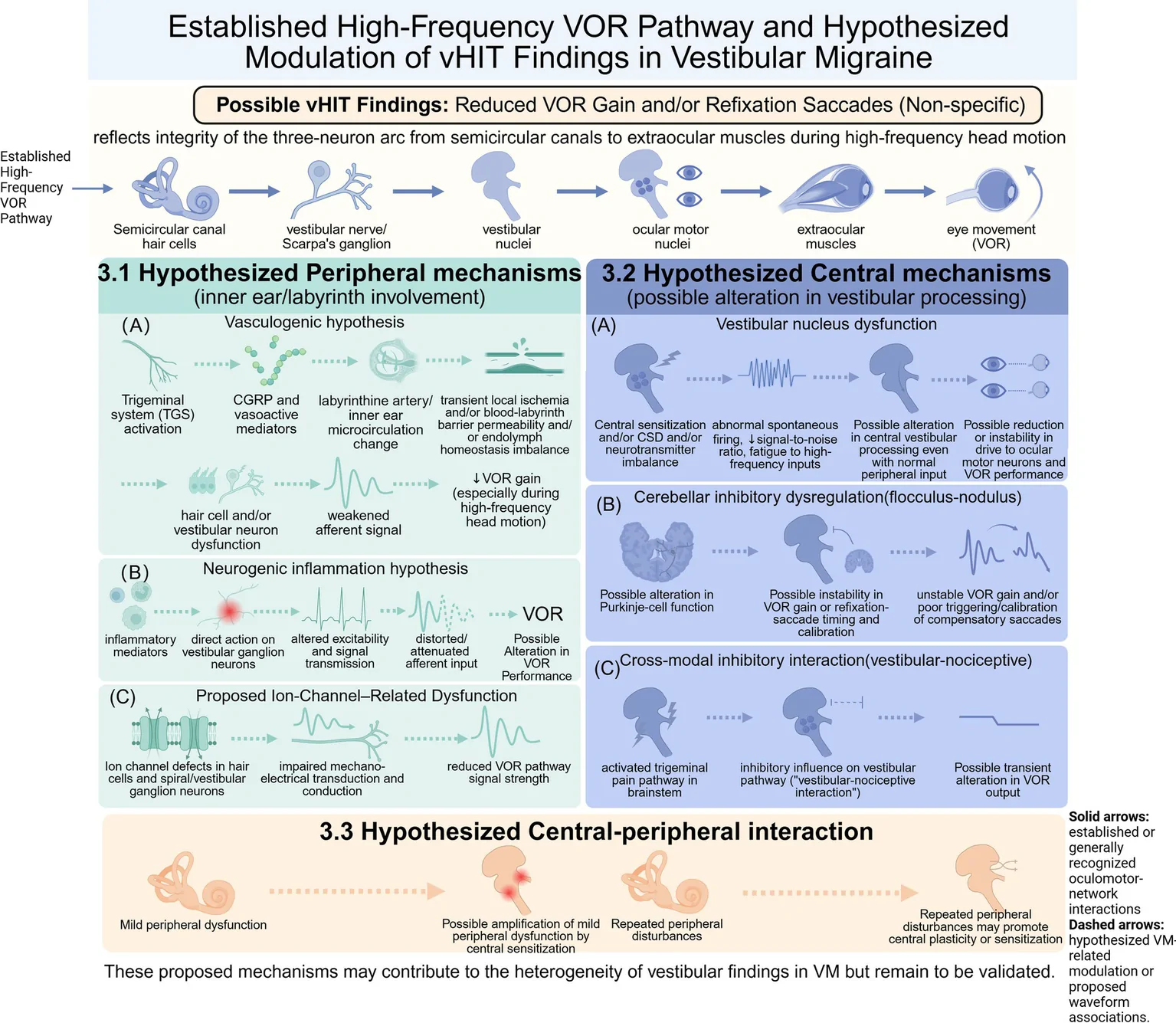
Established high-frequency vestibulo-ocular reflex pathway and hypothesized mechanisms potentially associated with abnormal Video Head Impulse Test findings in Vestibular Migraine. (The upper panel depicts the established high-frequency vestibulo-ocular reflex pathway from semicircular-canal hair cells through the vestibular nerve and Scarpa’s ganglion, vestibular nuclei, ocular motor nuclei, and extraocular muscles to compensatory eye movement. The lower panels summarize proposed mechanisms that may modulate this pathway in vestibular migraine. 3.1 Hypothesized peripheral modulation: Trigeminal-system activation and calcitonin gene-related peptide or other vasoactive mediators may influence inner-ear microcirculation, blood–labyrinth barrier permeability, endolymphatic homeostasis, hair-cell function, or vestibular-afferent signaling. Neurogenic inflammation and ion-channel-related dysfunction have also been proposed as possible contributors. 3.2 Hypothesized central modulation: Central sensitization, cortical spreading depression, neurotransmitter dysregulation, altered vestibular-nucleus processing, cerebellar modulation, and trigeminal–vestibular interactions may influence VOR performance or refixation-saccade timing and calibration. 3.3 Hypothesized central–peripheral interaction: Mild peripheral dysfunction may theoretically be amplified by central sensitization, whereas repeated peripheral disturbances may contribute to central plasticity. These proposed mechanisms are biologically plausible but have not been validated as direct causes of specific vHIT waveform morphologies in vestibular migraine. Reduced VOR gain and refixation saccades are non-specific findings and may also arise from peripheral vestibular dysfunction, compensation, biological variability, or technical artifacts. Solid arrows indicate established anatomical or physiological pathways, whereas dashed arrows indicate hypothesized mechanisms that remain to be validated.)

Preserved VOR gain should not be interpreted as evidence that vestibular structures or related neurochemical processes are normal. Rather, it indicates that high-frequency semicircular-canal VOR responses were preserved under the conditions of the vHIT examination. Neuroimaging studies have reported alterations in functional connectivity, cerebral perfusion, thalamic activity, and structural or functional measures in patients with VM (52–55); however, these observations do not establish a direct causal relationship with VOR gain or any specific vHIT waveform morphology. Although CACNA1A and ATP1A2 variants have been associated with selected migraine phenotypes (56–59), these genetic findings should not be generalized to typical VM or used to explain preserved VOR gain. Therefore, normal or near-normal vHIT gain neither confirms a specific central mechanism nor excludes dysfunction in other components of vestibular processing (60) (Figure 6).

Moreover, these results offer a reference for the differential diagnosis of VM. First, acute unilateral vestibulopathy/vestibular neuritis (AUVP/VN) is an acute vestibular syndrome characterized by the acute or subacute onset of sustained moderate-to-severe vertigo lasting at least 24 h, direction-fixed peripheral vestibular nystagmus, and unambiguous unilateral reduction of vestibulo-ocular reflex function, in the absence of acute central neurological, audiological, or otological symptoms or signs and when no alternative disorder better accounts for the presentation (3). A reduced VOR gain on vHIT may support the diagnosis, but no universal pathological cutoff has been established; a gain below 0.7 is only a working approximation and should be interpreted according to device- or laboratory-specific reference values (61–63). Second, Ménière’s disease (MD) is clinically characterized by recurrent vertigo episodes accompanied by fluctuating sensorineural hearing loss, tinnitus, or aural fullness. VOR gain on vHIT may remain preserved, particularly during earlier or interictal stages, while caloric weakness may coexist; reduced VOR gain may also occur depending on the stage and extent of vestibular involvement (34, 64). These overlapping findings limit the value of VOR gain as a standalone discriminator between MD and VM.

Accordingly, the principal clinical value of VOR gain lies in characterizing high-frequency semicircular-canal function and contributing to multimodal differential assessment, rather than in demonstrating structural preservation or a specific pathophysiological mechanism of VM.

### 4.2 Frequent occurrence of Refixation saccades

Several studies have reported refixation saccades in patients with vestibular migraine (VM), but estimates of their frequency and phase-dependent characteristics vary across study designs and populations. Koç et al. (47) observed refixation saccades in 52.3% of patients examined during a VM attack and in 10.2% of healthy controls; all saccades observed in the control group were covert. In a repeated-measures study of patients with definite VM, Batinović et al. (50) found no significant difference in the presence of refixation saccades between ictal and interictal examinations. Du et al. (39) described predominantly small-amplitude, bilateral, and scattered saccades, particularly in association with lateral semicircular-canal impulses, in cohorts including patients with VM and probable VM. Taken together, these studies indicate that refixation saccades can occur in VM, including during acute attacks, but they do not establish a consistent phase-dependent or VM-specific pattern. These findings should therefore be interpreted as non-specific adjunctive data in relation to VOR gain, diagnostic certainty, examination timing, recording quality, and the broader clinical context (39, 47, 50).

The following anonymized clinical cases are presented solely for illustrative purposes to demonstrate how selected video head impulse test and subjective visual vertical findings may be interpreted within the broader clinical context. They were not systematically enrolled, do not constitute a case series or an original study cohort, and were not included as evidence in the literature synthesis. All general statements and conclusions in the following subsections are derived from the cited literature rather than from these individual cases. Accordingly, these examples should not be used to establish diagnostic specificity, causal mechanisms, or generalizable video head impulse test phenotypes of vestibular migraine.

#### 4.2.1 Illustrative clinical case: transiently reduced horizontal-canal VOR gain with overt refixation saccades

Acute reduction in VOR gain accompanied by overt refixation saccades may occur during vestibular migraine (VM) attacks and may reflect transient impairment of high-frequency vestibulo-ocular stabilization rather than a VM-specific clinical phenotype (47, 50). This pattern is characterized by a decrease in VOR gain, which may fall below the device-specific reference range, together with compensatory overt refixation saccades after the head impulse (8, 21, 31, 37). Trigeminal–vestibular interactions and transient changes in vestibular-nucleus excitability have been proposed as possible contributors to vestibular symptoms during VM attacks (17–20). However, current evidence does not demonstrate that this specific vHIT pattern results from a short-term reversible blockade of high-frequency signal transmission between semicircular-canal hair cells and primary vestibular neurons; peripheral vestibular hypofunction, vestibular compensation, biological variability, and technical artifacts should also be considered (8, 15, 31, 32). Thus, reduced VOR-mediated gaze stabilization may be accompanied by overt refixation saccades, but this combination should be interpreted as a non-specific finding within the broader clinical context rather than as evidence of a specific VM mechanism (8, 30, 32–34) (Figure 6). A marked unilateral reduction in VOR gain accompanied by overt refixation saccades should prompt consideration of acute unilateral vestibular hypofunction, including acute unilateral vestibulopathy/vestibular neuritis (AUVP/VN) (3). However, this vHIT pattern is not disease-specific and is insufficient by itself to establish AUVP/VN, which requires integration of the acute vestibular syndrome, spontaneous nystagmus, neurological and audiological findings, and exclusion of alternative diagnoses. The following case is presented to illustrate this differential diagnostic context rather than to define a characteristic vHIT phenotype or mechanism of VM.

In the presence of similar acute low-gain patterns, evaluating the temporal evolution of vestibular findings may aid differential diagnosis. To illustrate this diagnostic challenge, an anonymized clinical case from our center is presented. A 39-year-old woman developed sudden severe vertigo accompanied by nausea and vomiting following a common cold. During the attack phase, vHIT showed a reduced VOR gain of 0.40 in the left horizontal semicircular canal, with a flattened eye-velocity curve and sharp, high-amplitude overt refixation saccades occurring after the head impulse (Figure 1A). Considered in isolation, these findings could raise suspicion of acute unilateral vestibular hypofunction, including AUVP/VN, but would not be sufficient to establish a specific peripheral vestibular diagnosis. However, the recurrent association of the vertigo episodes with menstruation, a family history of vestibular migraine, and a long-standing history of motion sickness supported consideration of VM in the differential diagnosis. To assess the temporal evolution of the vestibular findings, vHIT was repeated during the interictal follow-up. The VOR gain of the left horizontal semicircular canal improved from 0.40 to 0.79, and the amplitude of the overt refixation saccades was substantially reduced (Figure 1B). At a subsequent follow-up, VOR gains and eye-movement traces across all bilateral semicircular canals were stable, and the previously observed overt refixation saccades had largely resolved (Figure 1C). These serial findings demonstrate that the vHIT abnormality was not stable over time and may reflect a dynamic change in vestibular function. However, temporal improvement does not by itself establish the underlying mechanism, confirm VM, or exclude AUVP/VN; serial testing should therefore be interpreted together with the complete clinical course and complementary vestibular findings.

#### 4.2.2 Illustrative clinical case: bilateral small-amplitude covert Refixation saccades with preserved VOR gain

Bilateral small-amplitude covert refixation saccades with preserved vestibulo-ocular reflex (VOR) gain have been reported during vestibular migraine (VM) attacks, but they should not be regarded as a characteristic or disease-specific sign of VM (38, 39, 47). Clinically, this pattern may appear paradoxical: VOR gain remains within the device-specific reference range, whereas small-amplitude, scattered covert refixation saccades are recorded bilaterally, particularly in the horizontal semicircular canals. Central sensitization and altered vestibular sensory processing are recognized components of migraine pathophysiology and may theoretically influence gaze-stabilization networks (40, 41). However, current evidence does not demonstrate that reduced gating inhibition of the vestibular nuclei by the locus coeruleus–noradrenergic pathway or a lowered thalamocortical sensory-integration threshold directly produces this specific vHIT pattern. Subclinical peripheral vestibular dysfunction, vestibular compensation, age-related variability, and technical artifacts should also be considered (15, 21, 31, 32, 34). Therefore, when reproducible on technically adequate recordings, bilateral small-amplitude covert refixation saccades with preserved VOR gain may provide adjunctive information, but they do not have unique diagnostic value or establish a specific central mechanism, as summarized conceptually in Figure 6.

To illustrate this pattern, an anonymized 45-year-old woman from our center presented with severe vertigo triggered by fatigue, accompanied by right-sided temporoparietal headache, nausea, and vomiting. Physical examination revealed left-beating horizontal nystagmus. Based solely on the acute vertigo and vestibular signs, differentiation from peripheral vestibular disorders such as Ménière’s disease (MD) was necessary. However, a careful review of her clinical history revealed menstrual migraine, severe motion sickness since childhood, and a familial predisposition to migraine. Her attacks were also frequently provoked by sleep deprivation and strong odors, a pattern consistent with migraine-related sensory sensitivity.

During the attack phase, video head impulse testing (vHIT) showed preserved VOR gains across all semicircular canals, while the eye-velocity traces demonstrated small-amplitude, scattered covert refixation saccades in the bilateral horizontal semicircular canals (Figure 2A). At a subsequent examination, VOR gains remained preserved, while the amplitude of the bilateral covert refixation saccades decreased and their distribution became more regular (Figure 2B). By the later follow-up, VOR gains remained stable and the previously observed bilateral refixation saccades had largely resolved (Figure 2C). The absence of substantial hearing loss or tinnitus was considered together with the migraine-related clinical history and other vestibular findings in the differential assessment with MD.

This illustrative case demonstrates a potentially dynamic pattern of bilateral small-amplitude covert refixation saccades with preserved VOR gain. However, the temporal evolution observed in a single case does not demonstrate a reduced central sensory threshold or establish the specificity of this pattern for VM. These findings should therefore be regarded as non-specific adjunctive observations and interpreted together with the clinical phenotype, complementary vestibular and audiological assessments, and the technical quality and reproducibility of the vHIT recording.

#### 4.2.3 Illustrative clinical case: unilateral high-amplitude overt refixation saccades with preserved VOR gain

Unilateral high-amplitude overt refixation saccades with preserved vestibulo-ocular reflex (VOR) gain may occasionally be observed during vestibular migraine (VM) attacks, but they should not be regarded as a distinct or VM-specific phenotype (39). Clinically, this pattern is characterized by preserved VOR gain accompanied by prominent, relatively regular overt refixation saccades, predominantly involving one horizontal semicircular canal. Transient alterations in peripheral vestibular input, vestibular-nucleus excitability, or central gaze-stabilization control may theoretically contribute to such a pattern during VM attacks. However, current evidence does not demonstrate that unilateral labyrinthine vasospasm, inflammatory exudation, or impaired vestibulocerebellar inhibition directly produces this specific vHIT waveform. Subclinical peripheral vestibular dysfunction, biological variability, gaze-fixation instability, and technical artifacts should also be considered (8, 15, 21, 32, 34). Therefore, preserved VOR gain indicates that marked high-frequency semicircular-canal hypofunction is less likely, but it does not determine whether the accompanying refixation saccades arise from a central, peripheral, compensatory, or technical mechanism, as summarized conceptually in Figure 6.

This pattern of preserved VOR gain with unilateral high-amplitude refixation saccades may complicate clinical interpretation because similar unilateral vestibular signs can occur in structural peripheral or central vestibular disorders. To illustrate this diagnostic challenge, an anonymized 62-year-old woman from our center presented with acute severe vertigo and spontaneous nystagmus and had a 20-year history of hypertension and diabetes mellitus. These unilateral vestibular manifestations required consideration of disorders such as vestibular schwannoma, brainstem or cerebellar infarction, and advanced Ménière’s disease.

Nevertheless, a detailed review of the medical history and re-evaluation of the video head impulse test (vHIT) findings supported an alternative clinical interpretation. On the one hand, the patient exhibited migraine-related features before and during the attack, including a visual aura described as shimmering water ripples in the visual field, photophobia, and phonophobia, without substantial hearing loss. On the other hand, vHIT showed preserved VOR gains across the tested semicircular canals, while the eye-velocity trace of the left horizontal semicircular canal demonstrated prominent, relatively regular, high-amplitude overt refixation saccades during head impulses (Figure 3A). Considered together, these findings supported the inclusion of vestibular migraine in the differential diagnosis but did not independently establish the diagnosis or exclude a structural peripheral or central vestibular disorder.

Refixation saccades have been reported in patients with vestibular migraine (VM) and probable VM even when vestibulo-ocular reflex (VOR) gain is preserved, although their frequency, amplitude, and distribution are variable and non-specific (39). This combination indicates that the presence of refixation saccades and the measured VOR gain do not necessarily change in parallel. Calcitonin gene-related peptide (CGRP)-related pathways may form part of the broader pathophysiological background of VM, and emerging observational and pooled therapeutic evidence suggests that monoclonal antibodies targeting CGRP may improve vestibular symptoms in some patients (65, 66). However, these therapeutic studies do not establish that CGRP release directly disturbs the inner-ear microenvironment, generates a specific vHIT waveform, or determines the temporal resolution of refixation saccades. In this illustrative case, follow-up vHIT showed that VOR gains remained preserved, whereas the previously observed unilateral high-amplitude overt refixation saccades were markedly attenuated and had largely resolved (Figure 3B). Thus, the recorded saccadic pattern was dynamic, but its temporal evolution does not by itself establish a fully reversible CGRP-mediated mechanism. Clinically, serial vHIT findings should therefore be interpreted as adjunctive observations together with the clinical course, examination timing, recording quality, and complementary vestibular and audiological findings.

Furthermore, available evidence provides a basis for considering the timing and differential diagnostic implications of refixation saccades in vestibular migraine (VM). On the one hand, serial video head impulse testing (vHIT) may show changes in saccade frequency, amplitude, or distribution between ictal and interictal examinations; however, current studies do not establish a consistent phase-dependent pattern or a universal decline after 24 h (39, 47, 50). On the other hand, these findings may aid differential diagnosis when interpreted together with vestibulo-ocular reflex (VOR) gain and complementary clinical data. First, in acute unilateral vestibulopathy/vestibular neuritis (AUVP/VN), head impulses toward the affected side commonly elicit reduced VOR gain together with overt and/or covert refixation saccades, although the pattern may vary with lesion severity, examination timing, and vestibular compensation (8, 61, 62). Second, in Ménière’s disease (MD), VOR gain may be preserved or reduced, and refixation saccades may occur on the affected side; fluctuating auditory symptoms and dissociation between caloric testing and vHIT provide additional differential information (38, 64, 67). Third, in vestibular schwannoma, VOR gain and refixation-saccade patterns are variable and may be influenced by the extent of vestibular involvement and compensation; unilateral auditory findings and imaging therefore remain essential for diagnosis (16, 68). Overall, refixation saccades should be regarded as non-specific adjunctive findings whose clinical value depends on examination timing, recording quality, VOR gain, auditory and neurological findings, and complementary vestibular or imaging assessments. Their temporal and organizational characteristics warrant further prospective investigation. The principal clinical, vHIT, SVV, and caloric-test features that may assist in the differential assessment of suspected VM during an acute vestibular episode, AUVP/VN, MD, and VS are summarized in Table 1. These patterns should be interpreted as overlapping tendencies rather than mutually exclusive diagnostic profiles.

**Table 1:** Multimodal differential assessment of suspected vestibular migraine during an acute attack and selected peripheral vestibular disorders.

Disorder	Key clinical context	vHIT VOR gain	Covert/overt refixation saccades	SVV	Caloric testing	Principal interpretive limitation
Suspected vestibular migraine during an acute attack	Recurrent vestibular episodes lasting 5 min–72 h, a current or previous history of migraine, and migraine features during vestibular episodes; diagnosis remains clinical	Often preserved within device-specific reference ranges; variable or transient canal-specific reductions may occur	Covert and/or overt saccades may occur despite preserved gain; bilateral small-amplitude or occasional unilateral prominent patterns have been reported, but none is VM-specific	Usually normal or variably deviated; phase-related changes may occur but are not sufficiently consistent for diagnosis	May be normal or show unilateral weakness; abnormal caloric responses with preserved vHIT may occur but are not specific to VM	No single vestibular test confirms VM; findings must be integrated with migraine history and exclusion of competing disorders
AUVP/VN	Acute or subacute onset of sustained moderate-to-severe vertigo lasting ≥24 h, with direction-fixed spontaneous peripheral vestibular nystagmus and no acute central neurological, audiological, or otological symptoms or signs; no alternative disorder better accounts for the presentation	Typically reduced during impulses toward the affected side in the involved canal or canals; horizontal-canal gain may remain normal in isolated inferior-division disease	Overt and/or covert saccades commonly occur during impulses toward the affected side; their latency and temporal clustering may evolve with vestibular compensation	Ipsilesional deviation toward the affected ear may occur, particularly with superior-division involvement; it may normalize during recovery and may be absent in isolated inferior-division disease	Unilateral caloric weakness is common when the horizontal canal/superior vestibular nerve is involved; caloric responses may remain normal in isolated inferior vestibular neuritis	Findings depend on the affected vestibular division, examination timing, and degree of compensation; diagnosis still requires exclusion of central and other peripheral vestibular disorders
Ménière’s disease	Two or more spontaneous vertigo episodes lasting 20 min–12 h, with audiometrically documented low- to medium-frequency sensorineural hearing loss and fluctuating aural symptoms in the affected ear; not better accounted for by another vestibular diagnosis	Often preserved, particularly between attacks, even when caloric weakness is present; may be reduced during attacks or in some patients with more extensive vestibular involvement	Refixation saccades may occur on the affected side, with or without reduced gain; they are not disease-specific	Variable; an ipsilesional deviation may occur, but its direction and magnitude depend on disease phase, otolith involvement, and compensation and are insufficiently specific to distinguish MD from VM	Unilateral caloric weakness is common; a reduced caloric response with relatively preserved horizontal-canal vHIT is a frequently reported dissociation, but is not diagnostic in isolation	Diagnosis relies primarily on the episodic audiovestibular history and audiometry; no isolated vHIT, SVV, or caloric–vHIT dissociation is diagnostic
Vestibular schwannoma	Asymmetric or unilateral sensorineural hearing loss, unilateral tinnitus, and/or other ipsilateral audiovestibular symptoms; diagnosis requires dedicated MRI of the internal auditory canals and cerebellopontine angles	Variable; may be normal or reduced in one or more canal planes according to vestibular-nerve involvement, tumour characteristics, and compensation; normal vHIT does not exclude VS	Covert and/or overt refixation saccades may occur during impulses toward the affected side when high-frequency VOR function is impaired; they are not disease-specific	May be abnormal, but its direction and magnitude are variable and show no sufficiently consistent relationship with tumour side or size to support diagnosis	Unilateral caloric weakness is common and may be present despite normal vHIT; caloric testing and vHIT provide complementary low- and high-frequency information	Vestibular tests cannot establish VS; asymmetric audiological findings and dedicated internal auditory canal MRI remain central to diagnosis

AUVP/VN, acute unilateral vestibulopathy/vestibular neuritis; MD, Ménière’s disease; MRI, magnetic resonance imaging; SVV, subjective visual vertical; vHIT, video head impulse test; VM, vestibular migraine; VOR, vestibulo-ocular reflex; VS, vestibular schwannoma. The patterns summarized in this table represent commonly reported tendencies rather than disease-specific diagnostic criteria. Results may vary according to attack phase, lesion extent, vestibular compensation, testing protocol, device-specific reference ranges, and recording quality (1–3, 76–82).

Accordingly, the main value of this structured comparison is not to assign a diagnosis from an isolated vestibular-test result, but to identify concordant or discordant patterns that guide the subsequent clinical, audiological, neurological, and imaging evaluation.

### 4.3 Adjunctive role of subjective visual vertical and complementary otolith assessment

When interpreting video head impulse test (vHIT) findings in patients with suspected vestibular migraine (VM), the subjective visual vertical (SVV) test may provide complementary information on graviceptive perception that is not captured by high-frequency semicircular-canal assessment alone. These two examinations evaluate different components of vestibular function and may therefore contribute to a more comprehensive clinical assessment, although neither can independently establish the diagnosis of VM. During SVV testing, the patient adjusts a visible line to the perceived gravitational vertical in the absence of external visual references, and the angular difference between the adjusted line and the true vertical is recorded as the SVV deviation angle (50, 67, 69). SVV reflects the integrated processing of graviceptive information arising from the otolith pathways together with central vestibular, visual, and somatosensory contributions, rather than serving as an isolated measure of utricular function (70). Depending on the testing paradigm, SVV may be assessed under static or dynamic conditions, and a deviation within approximately ±2.5° is commonly used as a reference range in healthy adults, although normative values may vary with the testing method and population (69, 71, 72).

Because SVV primarily measures perceptual verticality, an abnormal SVV result should not be regarded as a complete assessment of peripheral otolith-organ function. Accordingly, SVV should not be used as the sole basis for characterizing otolith-related dysfunction. Cervical and ocular vestibular-evoked myogenic potentials (cVEMPs and oVEMPs) provide complementary reflex-based information that predominantly samples the saccular–vestibulocollic and utricular–vestibulo-ocular pathways, respectively, although the relative contributions of the otolith organs depend on the stimulus and recording paradigm (5). In patients with suspected vestibular migraine, VEMPs may therefore be considered when otolith-related dysfunction is clinically suspected or when abnormal SVV findings require further clarification. However, VEMP abnormalities in vestibular migraine are variable and may overlap with those observed in Ménière’s disease and other vestibular disorders; no individual VEMP measure has been validated as a diagnostic biomarker for vestibular migraine (1, 73). Thus, combining perceptual and reflex-based vestibular assessments may improve characterization of the involved vestibular pathways without converting any single test into an independent diagnostic marker for vestibular migraine. Previous studies have reported phase-related changes in subjective visual vertical (SVV) findings in patients with vestibular migraine (VM). For example, Batinović et al. (50) found that the mean SVV deviation was 1.58° during the acute phase and 0.65° during the symptom-free phase, indicating a greater disturbance of perceived verticality during attacks. When considered together with attack-related symptoms such as nausea, photophobia, and phonophobia, this temporal difference may reflect dynamic changes in graviceptive perception and multisensory spatial orientation. However, the magnitude of SVV deviation varies among patients, and an abnormal SVV result is neither necessary nor specific for the diagnosis of VM (50, 75, 83). SVV may therefore complement video head impulse test (vHIT) findings by assessing a component of vestibular perception that is not directly represented by high-frequency semicircular-canal VOR gain.

SVV may also provide adjunctive information in the differential assessment of VM and Ménière’s disease (MD). Previous studies have described abnormal SVV findings in both disorders, although their direction, magnitude, and temporal characteristics may differ according to disease phase, peripheral vestibular involvement, central compensation, and the testing paradigm (70, 75). In MD, an ipsilesional SVV tilt may occur in association with asymmetric peripheral otolith input, whereas SVV abnormalities in VM may reflect variable disturbance of graviceptive and multisensory integration. Nevertheless, these patterns overlap and should not be used independently to distinguish VM from MD. Thus, preserved vHIT gain accompanied by abnormal SVV may identify discordance between high-frequency semicircular-canal function and graviceptive perception, but it does not by itself establish VM. Its clinical value lies in prompting integrated interpretation with migraine-related history, auditory symptoms, VEMPs, caloric testing, and other vestibular findings. An illustrative clinical case of abnormal subjective visual vertical (SVV) with preserved video head impulse test (vHIT) findings involved an anonymized 62-year-old woman from our center. The patient experienced recurrent episodes of dizziness accompanied by occasional headache and sudden falls. Her symptoms were aggravated in the supine position, during neck extension or rotation, and by emotional fluctuations. Because substantial hearing loss was not evident and cervical range-of-motion assessment showed restricted neck extension and rotation, the positional symptoms could initially raise consideration of cervicogenic dizziness or other position-related vestibular disorders.

Further vestibular assessment, however, showed discordant findings. On the one hand, vHIT demonstrated preserved vestibulo-ocular reflex (VOR) gains and stable eye-velocity traces in the bilateral horizontal semicircular canals, without evident refixation saccades. These findings suggested preserved high-frequency horizontal semicircular-canal function but did not independently exclude peripheral vestibular or other structural disorders. On the other hand, SVV testing showed a marked deviation of −7.75° (Figure 4). This abnormality indicated impaired graviceptive perception or spatial-orientation processing, but SVV alone could not localize the dysfunction specifically to the left utricle or distinguish a peripheral otolith abnormality from altered central multisensory integration.

When interpreted together with the patient’s clinical history, symptom triggers, cervical assessment, and other vestibular findings, the discrepancy between preserved vHIT findings and abnormal SVV supported a broader evaluation of vestibular migraine (VM) rather than attribution of the symptoms solely to cervical restriction or fixed horizontal semicircular-canal loss. However, this single illustrative case does not establish that the SVV deviation was caused by a specific central mechanism of VM. Its clinical value lies in demonstrating that preserved high-frequency semicircular-canal function may coexist with abnormal graviceptive perception and that such discordant findings should prompt integrated assessment using clinical diagnostic criteria and complementary vestibular examinations.

Based on the preceding analysis, refixation saccades may provide information that is not fully captured by vestibulo-ocular reflex (VOR) gain during the acute phase of vestibular migraine (VM), but neither parameter has sufficient specificity to establish the diagnosis independently. Subjective visual vertical (SVV) and vestibular-evoked myogenic potentials (VEMPs) may provide complementary information on graviceptive perception and otolith-related reflex pathways, respectively. Nevertheless, abnormalities in these assessments are variable and non-specific and should be interpreted together with the established clinical diagnostic criteria, detailed history, recording quality, auditory and neurological findings, and other complementary vestibular examinations. Another potentially complementary clinical observation is pupillary hippus—termed “pupillary nystagmus”by Gufoni and Casani—characterized by repetitive fluctuations in pupil diameter under relatively constant illumination. A comparative study published in 2023 reported that pupillary hippus was frequently observed during the interictal phase in patients with definite vestibular migraine and proposed it as a possible objective clinical sign (74). A subsequent case–control study using infrared pupillometry further demonstrated higher Pupillary Unrest Activity Level values in patients with vestibular migraine than in healthy controls (84). These findings support further investigation of pupillary dynamics as an objective adjunctive measure. However, pupillary hippus may also occur physiologically or in association with autonomic and neurological conditions, and the available studies used different control populations and involved overlapping investigator groups. Moreover, neither study established an acute-attack-specific finding or independently validated its ability to distinguish vestibular migraine from major peripheral vestibular disorders. Pupillary hippus should therefore be regarded as a promising adjunctive sign rather than the only objective sign or a validated stand-alone biomarker for vestibular migraine. Its incremental diagnostic value beyond the established clinical criteria and complementary vestibular assessments requires confirmation in independent, multicenter cohorts.

## 5 Conclusion

Video head impulse testing (vHIT) may contribute to the differential assessment of patients presenting with acute vertigo or dizziness by characterizing high-frequency semicircular-canal vestibulo-ocular reflex (VOR) function and identifying refixation saccades. In patients with acute vestibular migraine (VM), VOR gain is often preserved, although transient gain reduction, overt or covert refixation saccades, and subjective visual vertical (SVV) abnormalities may be observed in some patients. These findings are not specific to VM and may also be influenced by peripheral vestibular hypofunction, vestibular compensation, biological variability, gaze-stabilization abnormalities, examination timing, or technical artifacts. Therefore, they should not be used independently to establish the diagnosis of VM, exclude structural vestibular disease, or infer a particular central pathophysiological mechanism.

The potential clinical value of vHIT lies in the integrated interpretation of VOR gain, the timing, amplitude, distribution, and reproducibility of refixation saccades, recording quality, and changes across serial examinations. These findings should be considered together with the detailed clinical history, migraine-related features, auditory and neurological findings, and complementary vestibular assessments. SVV and vestibular-evoked myogenic potentials (VEMPs) may provide additional information on graviceptive perception and otolith-related reflex pathways, but their abnormalities are also variable and non-specific. The illustrative clinical cases presented in this review demonstrate possible approaches to interpreting discordant or evolving vestibular findings; they do not constitute evidence for generalizable VM-specific vHIT phenotypes.

Future prospective studies with adequate sample sizes, standardized acquisition and reporting procedures, rigorous artifact control, and repeated assessments during ictal and interictal phases are needed to determine whether particular vHIT, SVV, or complementary vestibular parameters provide reproducible diagnostic or prognostic information in VM. Ultimately, these tests should be incorporated into an integrated assessment framework based on established clinical diagnostic criteria rather than developed as stand-alone biomarkers. Such an approach may improve the differential evaluation of acute vestibular presentations while preserving the fundamentally clinical basis of VM diagnosis.
